# Inhibition of Angiogenesis by MiR-524-5p through Suppression of AKT and ERK Activation by Targeting CXCR7 in Colon Cancer Cells

**DOI:** 10.1155/2022/7224840

**Published:** 2022-11-10

**Authors:** Xiang Li, Zitao Li, Caijuan Li, Yi Fang, Gaosen Zhang, Yudie Yan, Xiaodi Zhao, Xiaole Song, Zhen Zhang

**Affiliations:** ^1^Department of Ultrasonic Diagnosis, The First Hospital of China Medical University, 155 Nanjing North Street, Shenyang, Liaoning, China; ^2^Department of Orthopedic Surgery, Hongqi Hospital, Mudanjiang Medical University, 5 Tongxiang Road, Mudanjiang, Heilongjiang, China; ^3^Department of Ultrasound, Hongqi Hospital, Mudanjiang Medical University, 5 Tongxiang Road, Mudanjiang, Heilongjiang 157011, China

## Abstract

Increasing evidence shows that alterations in microRNA (miRNA) expression are involved in the occurrence and development of various malignant tumors, including colon cancer. MiRNA-524-5p has been reported to have anticancer activity in colon cancer. This study explored the influence of the miRNA-524-5p/CXCR7 axis on angiogenesis using colon cancer cells and further studied the mechanisms involved. We found that changing the expression of miRNA-524-5p can affect colonic proliferation, migration, and angiogenesis. Furthermore, angiogenesis induced by miRNA-524-5p overexpression was reversed by overexpression of CXCR7 in HT-29 cells, while the opposite was observed in Caco-2 cells. Furthermore, miRNA-524-5p inhibited the activation of AKT and ERK signaling by targeting CXCR7. Overall, our results indicated that the miRNA-524-5p/CXCR7 axis regulated angiogenesis in colon cancer cells through the AKT and ERK pathways.

## 1. Introduction

Colorectal cancers (CRCs) are the third most common malignancy in the world [1, 2]. In the past 30 years, the global incidence and mortality due to colon cancer have been high [3]. Although trends in the incidence and mortality of colorectal cancer vary between countries, the global burden of this disease is projected to increase over the next decade [1]. Therefore, there is an urgent need to study new targeting factors in colon cancer. There are several studies showing that the inhibition of blood vessel formation can play an important role in cancer progression [4, 5]. Many factors are involved in angiogenesis, such as vascular endothelial growth factor (VEGF). Most tumors are associated with the overexpression of VEGF, especially the VEGF-A/VEGFR2 axis, which plays a key role in angiogenesis [6]. Therefore, it is very important to better understand the mechanism of angiogenesis-related factors in colon cancer.

MicroRNA (miRNA) are short RNA molecules of 19–25 nucleotides in size that can silence target genes after transcription [7, 8]. A single miRNA can target hundreds of mRNAs and affect the expression of many genes involved in various interaction pathways [9–11]. In nonsmall cell lung cancer (NSCLC), the knockdown of LINC00184 inhibits cell proliferation, migration, and accelerates apoptosis, which are closely related to the regulation of the miR-524-5p/HMGB2 axis [12]. Zhao et al. [13] showed that miR-524 has an inhibitory effect on glioma cells and targets C-myc, which binds to its promoter region and activates the expression of the epidermal growth factor receptor (EGFR). The high expression of LncRNA TUG1 in oral squamous cell carcinoma (OSCC) mediates the expression of distal homeobox 1 (DLX1) through competitive binding to miR-524-5p [14]. Therefore, dysregulation of miRNA-524-5p can be observed in a wide range of diseases, including colon cancer [15]. However, its molecular mechanism for promoting angiogenesis in colon cancer needs further study.

Growing clinical data on colon cancer suggests that mRNA and protein levels of the CXC chemokine receptor 7 (CXCR7) were up-regulated in tumor tissues of colon cancer patients with lymph node metastases compared to non-metastatic tumors [16]. CXCR7 is attached to chemokine-specific seven transmembrane guanosine-bindingprotein-coupled receptors [17]. Several studies suggested that, except for embryonic neurons, fetal heart tissues, and certain hematopoietic cells, CXCR7 is absent in most normal tissues in humans but is highly expressed in the human endometrium and several types of malignancies, including colon cancer [18–20]. This new chemokine receptor is defined as a high-affinity receptor for the CXC chemokine ligand 12 (CXCL12) that can also bind CXCL11 [21]. Increasing CXCR7 expression accelerated the growth and metastasis capacity of various malignant tumors, which was accompanied by the regulation of angiogenesis and immunity [22]. Therefore, to study the molecular mechanism of miR-524-5p that regulates CXCR7 expression in colon cancer, we respectively regulated the expression of miR-524-5p and CXCR7 in HT-29 and Caco-2 cells to evaluate the influence of the miR-524-5p/CXCR7 axis on colon cancer angiogenesis.

## 2. Material and Methods

### 2.1. Cell Culture

HCoEpiC, SW480, HCT116, Caco-2, RKO, HT-29 cell lines, and human umbilical vein endothelial cells (HUVEC) were purchased from the Cell Bank of the Chinese Academy of Sciences (Shanghai, China). The cell lines HCoEpiC, HCT116, RKO, HT-29, and HUVECs were cultured in PRMI 1640 medium (HyClone, GE Healthcare, UK) containing 10% fetal bovine serum (FBS, HyClone). The Caco-2 and SW480 cell lines were cultured in DMEM medium containing 10% fetal bovine serum. All cells were cultured at 37°C in a 5% CO_2_ atmosphere.

### 2.2. Transfection

HT-29 cell lines at 70% confluence, were prepared for transfection with the pCMV6 entry CXCR7 plasmid (CXCR7OE, OriGene, WuXi, China). HT-29 cells were grown in a transfection medium containing miRNA-524-5p mimic (RiboBio, Guangzhou, China) and CXCR7OE at 37°C for 48 h while Caco-2 cells were grown in a transfection medium containing the miRNA-524-5p inhibitor (RiboBio) and CXCR7 siRNA (RiboBio) at 37°C for 48 h. CXCR7siRNA sequences were 5′‐GGAAGAUCAUCUUCUCCUATT‐3′ (sense) and 5′‐UAGGAGAAGAUGAUCUUCCGG‐3′ (antisense). Cells were transfected with Lipofectamine 3000 (Invitrogen, Grand Island, NY) according to the manufacturer's instructions. After transfection, HT-29 and Caco-2 cells were harvested for the future experiments.

### 2.3. CCK-8 Assay

HT-29 and Caco-2 cells were seeded in 96-well plates at a density of 4 × 10^3^ cells. Cell growth of HT-29 cells, transfected with the miRNA-524-5p mimic, and Caco-2 cells, transfected with the miRNA-524-5p inhibitor, was tested using the CCK-8 kit (Dojindo, Kumamoto, Japan) after 0, 24, 48, and 72 hours of incubation. Next, HT-29 cells cotransfected with miRNA-524-5p mimic and CXCR7OE, and Caco-2 cells, cotransfected with miRNA-524-5p inhibitor and CXCR7 siRNA, were cultured for 48 hours, and then cell proliferation was measured using the CCK-8 kit. A microplate reader was used to measure the optical density (OD) at a wavelength of 450 nm.

### 2.4. The 5-Ethynyl-20-Deoxyuridine (EdU) Assay

HT-29 and Caco-2 cells were seeded in a 24-well plate at a density of 2 × 10^5^ cells per well and cultured in the normal growth stage. Cells were transfected with the miRNA-524-5p mimic and the miRNA-524-5p inhibitor for 48 hours, then 50 *μ*M EdU was added, and cells were incubated at 37°C for 2 hours. Subsequently, cells were stained using cell light EdU DNA imaging (RiboBio).

### 2.5. Cell Migration Assay

The transfected cells (4 × 10^5^ cells/well) described above were seeded in the lower chamber and cultured until the cells adhered to the plate. HUVEC (2 × 10^5^ cells/well) was seeded in the upper chamber and incubated at 37°C for 12 h. After fixation and staining, the migrating cells were photographed and counted using a microscope.

### 2.6. Tube Formation Assay

A 200 *μ*l volume of Matrigel (BD Biosciences) was added to each well in the lower chamber of the 24-well plate and incubated at 37°C for 30 minutes. HUVEC was seeded in the upper chamber at 2 × 10^5^ cells per well. Subsequently, the transfected cells (4 × 10^5^ cells/well) were resuspended in 200 *μ*L of complete medium and added to the upper chamber. After incubation for 6 hours at 37°C, the number of junctions was counted after images were acquired with a microscope.

### 2.7. Enzyme-Linked Immunosorbent Assay (ELISA)

An ELISA kit (R&D Systems) was used to estimate the concentrations of CXCL11, CXCL12, and VEGF according to the manufacturer's instructions. The OD value at 450 nm was measured using a microplate reader.

### 2.8. Dual-Luciferase Reporter Assay

The binding site of miRNA-524-5p and CXCR7 was predicted by the TargetScan bioinformatics website (https://www.targetscan.org/). HT-29 and Caco-2 cells were cotransfected with miRNA-524-5p mimic or negative control miRNA and a CXCR7 3′-UTR wild-type (WT) or CXCR7 3′-UTR mutant (MUT) reporter plasmid (RiboBio) using Lipofectamine 3000 (Invitrogen) under the manufacturer's guidance to verify whether CXCR7 was a direct miRNA-524-5p target gene. The Dual-Luciferase Reporter Gene Detection Kit (KeyGENBioTECH, China) was used to check luciferase activity.

### 2.9. Tumor Model

BALB/c nude mice (aged 4–5 weeks) were obtained from Beijing Vital River Laboratory Animal Technology Co., Ltd. After one week of adaptation, BALB/c nude mice were injected with HT-29 cells to establish a subcutaneous human colon cancer xenograft model. When the tumors grew to approximately 0.5 cm^3^ in volume, miR-524-5p agomir and the negative control agomir were injected into the tumor every three days. The tumor size was measured every 7 days. Animal studies were performed in compliance with the Guide for the Care and Use of Laboratory Animal Resources (1996), the National Research Council, and approved by the Animal Ethics Committee of the China Medical University (IACUC Issue No. 16071). All procedures were followed under supervision and inspection by the Committee and the Laboratory Animal Department.

### 2.10. Real-Time Polymerase Chain Reaction Analysis

After the total RNA of the sample was extracted with TRIzol reagent (Invitrogen, Grand Island, NY, USA), real-time polymerase chain reaction (RT-PCR) was performed with a reverse transcription kit (TaKaRa, Dalian, China). The primers used were as follows:5′-GTTGGCTCTGGTGCAGGGTCCGAGGTATTCGCACCAGAGCCAACGAGAAA-3′ (miR-524-5p RT), 5′-CGCTACAAAGGGAAGCACTT-3′ (miR-524-5p forward), 5′-GCAGGGTCCGAGGTATTC-3′ (miR-524-5p reverse); 5′-GCTTCGGCAGCACATATACT-3′ (U6 forward), 5′-GCAGGGTCCGAGGTATTC-3′ (U6 reverse); 5′-CAACCTCTTCGGCAGCATTT-3′ (CXCR7 forward), 5′-ACGACACGGCGTACCATCTT-3′ (CXCR7 reverse); 5′-TCACCAAGGCCAGCACATAG-3′ (VEGFA forward), 5′-AGGCTCCAGGGCATTAGACA-3′ (VEGFA reverse); 5′-GACCTGACCTGCCGTCTAG-3′ (GAPDH forward), 5′-AGGAGTGGGTGTCGCTGT-3′ (GAPDH reverse).

### 2.11. Western Blotting Analysis

RIPA Lysis Buffer (Beyotime, Shanghai, China) was used to lyse the protein from cells or tissue samples. Proteins were then electrophorized on a 10% or 8% SDS-PAGE gel. After electrophoresis, the protein was transferred to a polyvinylidene fluoride (PVDF) membrane (Millipore, USA), blocked in 5% skim milk powder for 2 hours, and incubated with the diluted antibody overnight at 4°C. The primary antibodies used were as follows: AKT, p-AKT Ser473 (Cell Signaling Technology, Danvers, Mass, USA), ERK, p-ERKThr202/Tyr204, PDGF (Abcam, Cambridge, UK), GAPDH, E2F1, VEGF, and CXCR7 (Proteintech, China). The protein bands were detected using ImageLab software and displayed using photographic film.

### 2.12. Immunohistochemical Analysis

The tumor tissues obtained were fixed, embedded, and sliced (thickness, 6 *μ*m) and then subjected to immunohistochemical experiments. Tumor sections were incubated with the primary antibodies Ki67, CXCR7, AKT, p-AKT, ERK, p-ERK, VEGF, PDGF, and CD34 at 4°C overnight, and then incubated with secondary antibodies. The sections were stained with diaminobenzidine. Immunopositive proteins were observed under an optical microscope at 200x and 400x magnification.

### 2.13. Statistical Analysis

All data were expressed as means ± standard deviation (SD). All statistical analyses were performed using SPSS version 20.0 software (IBM Corp., Armonk, NY, USA). Statistical differences between groups were calculated using one‐way analysis of variance or the Student's *t*-test. The *p*-value ＜0.05 was considered statistically significant.

## 3. Results

### 3.1. MiR-524-5p Influenced Cell Proliferation

**Figure 1:**
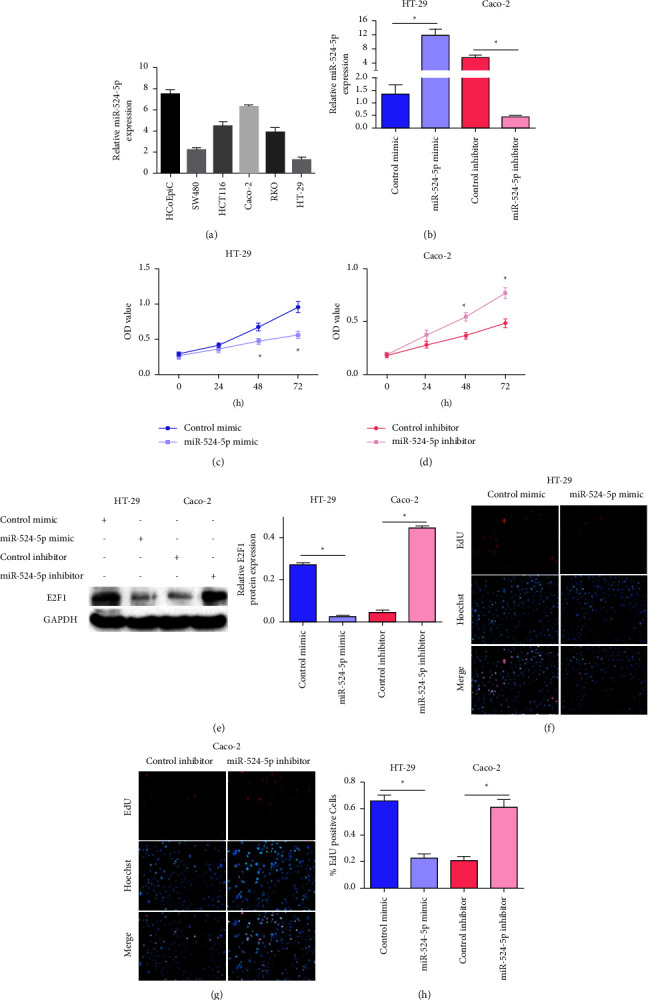
Effects of miR-524-5p on proliferation. (a) The relative miR-524-5p expression in colon cancer cells. (b) Relative miR-524-5p expression in HT-29 and Caco-2 cells after relevant transfection. CCK8 assay showing the proliferation of HT-29 (c) and Caco-2 (d) cells after the indicated transfection. (e) Relative E2F1 protein expression in HT-29 and Caco-2 cells after relevant transfection. Representative images of EdU-positive cells of HT-29 (f) and Caco-2 (g) cells after the indicated transfection. (h) Quantitative measurement of EdU-positive cells. Magnification 200x. *∗p* < 0.05 vs. the indicated group.

To investigate the role of miR-524-5p in colon cancer proliferation, we measured miR-524-5p expression in different colon cancer cell lines (HCoEpiC, SW480, HCT116, Caco-2, RKO, and HT-29). MiR-524-5p expression was the lowest in the HT-29 cell line and the highest in Caco-2 cells (Figure 1(a)). Subsequently, we used miR-524-5p mimic to induce miR-524-5p overexpression in HT-29 cells and miR-524-5p inhibitors to decrease miR-524-5p expression in Caco-2 cells (Figure 1(b)). The CCK-8 cell growth assay showed that the miR-524-5p mimic significantly reduced the proliferation of HT-29 cells (Figure 1(c)). In contrast, the miR-524-5p inhibitor significantly increased Caco-2 cell proliferation (Figure 1(d)). Furthermore, we further verified the expression of E2F1 in HT-29 and Caco-2 cells, and the results showed that miR-524-5p mimic reduced E2F1 expression in HT-29 cells. The miR-524-5p inhibitor up-regulated E2F1 expression in Caco-2 cells (Figure 1(e)). The EdU assay showed that the number of EdU-positive cells in HT-29 cells transfected with miR-524-5p mimic was significantly reduced. In contrast, the number of EdU-positive cells in Caco-2 cells transfected with miR-524-5p inhibitor significantly increased (Figures 1(f)–1(h)).

### 3.2. Effects of MiR-524-5p on the Migration and Tube Formation of HUVECs

**Figure 2:**
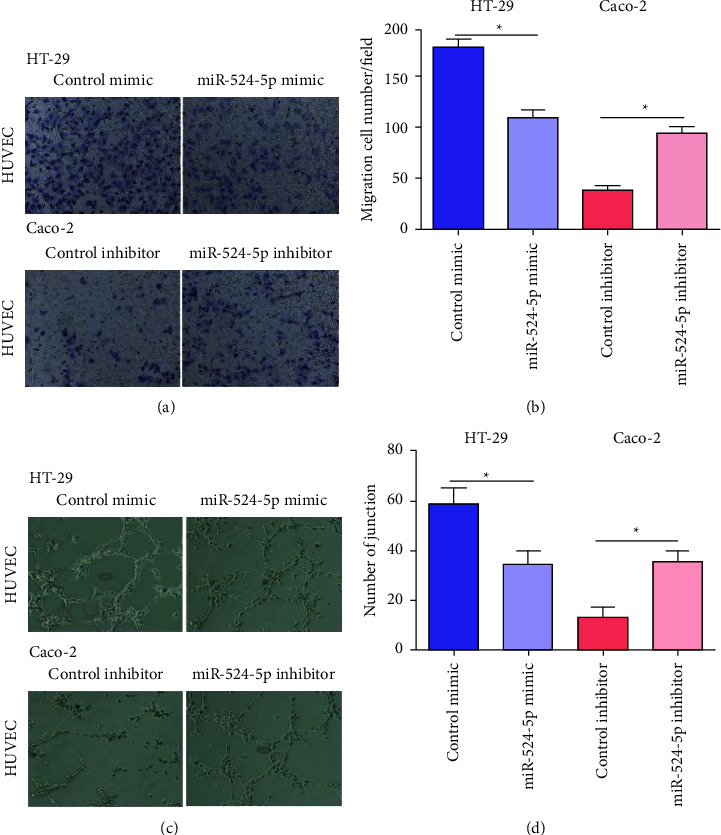
Effects of miR-524-5p on the migration and tube formation of HUVECs. (a) Representative images of migration of HT-29 and Caco-2 cells after relevant transfection. (b) Quantitative measurement of migration cell number. (c) Representative image of tube formation in HT-29 and Caco-2 cells after relevant transfection. (d) Quantitative measurement of the junction number. Magnification 200x. *∗p* < 0.05 vs. the indicated group.

To explore whether miR-524-5p is associated with tumor angiogenesis, we cocultured colon cancer cells transfected with miR-524-5p with HUVECs and used transwell and lumen formation experiments to detect the migration and angiogenesis abilities of HUVECs. As shown in Figures 2(a) and 2(b), the number of migrating cells in cocultured HUVECs and HT-29 cells transfected with miR-524-5p mimic decreased significantly. The number of cocultured HUVECs with migrating cells and Caco-2 cells transfected with the miR-524-5p inhibitor increased significantly. After coculturing with HT-29 cells transfected with the miR-524-5p mimic, the number of HUVECs junctions was significantly reduced. In contrast, coculturing of Caco-2 cells transfected with the miR-524-5p inhibitor significantly increased the number of HUVECs junction (Figures 2(c) and 2(d)).

### 3.3. MiR-524-5p Influences VEGF Expression

**Figure 3:**
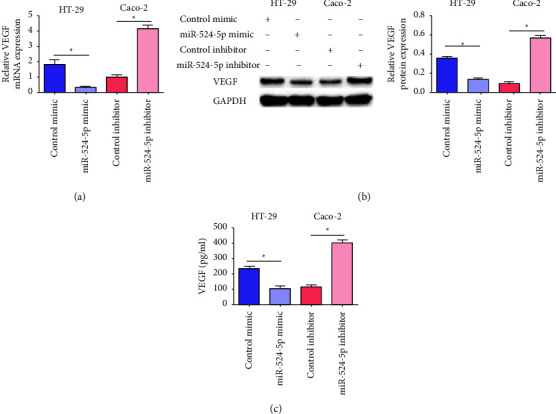
Effects of miR-524-5p on VEGF expression. (a) The relative mRNA expression of VEGF in HT-29 and Caco-2 cells. (b) The relative protein expression of VEGF in HT-29 and Caco-2 cells. (c) The concentration of VEGF in HT-29 and Caco-2 cells. ^*∗*^*p* < 0.05 vs. the indicated group.

Since VEGF is an important factor involved in angiogenesis, we detected the expression of VEGF in HT-29 and Caco-2 cells transfected with miR-524-5p by quantitative RT-PCR and western blotting. As shown in Figures 3(a) and 3(b), the expression of VEGF mRNA and protein in HT-29 cells transfected with miR-524-5p mimic was significantly reduced, and the expression of VEGF mRNA and protein in Caco-2 cells transfected with the miR-524-5p inhibitor increased significantly. At the same time, we also detected the concentration of VEGF in the supernatant of each group. The results showed that the concentration of VEGF in HT-29 cells transfected with miR-524-5p mimic was significantly reduced, while the concentration of VEGF in Caco-2 cells transfected with the miR-524-5p inhibitor was significantly increased (Figure 3(c)).

### 3.4. CXCR7 Acted as a Target of miR-524-5p

**Figure 4:**
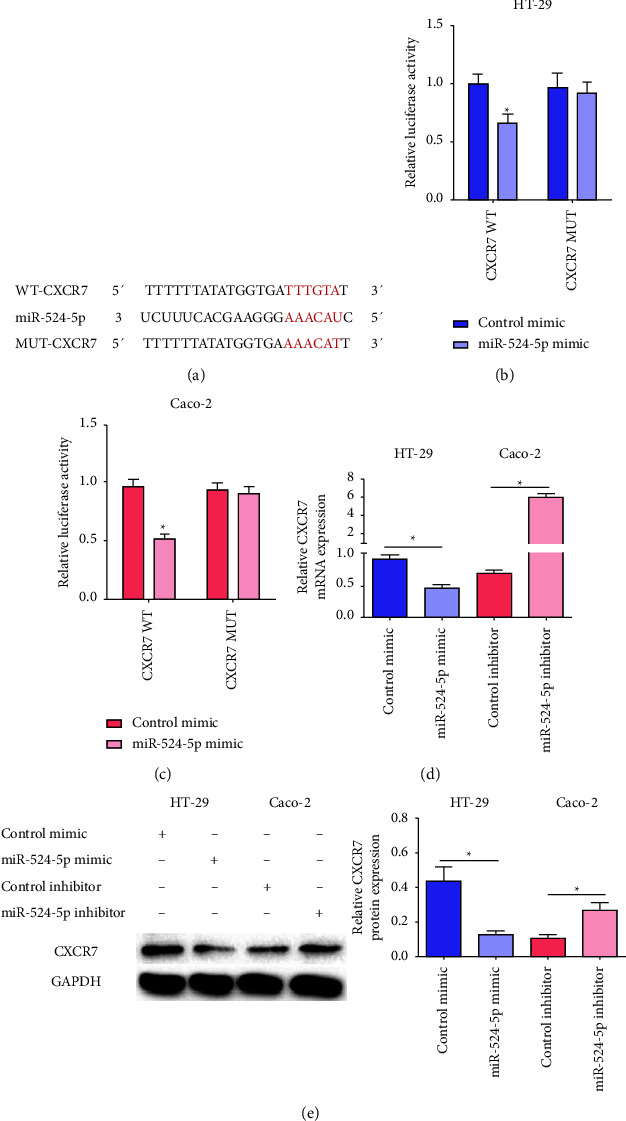
CXCR7 acts as a target of miR-524-5p. (a) The binding region between miR-524-5p and CXCR7. (b, c) A dual-luciferase reporter assay to test the luciferase activity in HT-29 and Caco-2 cells. (d) The relative mRNA expression of CXCR7 in HT-29 and Caco-2 cells. (e) The relative protein expression of CXCR7 in HT-29 and Caco-2 cells. ^*∗*^*p* < 0.05 vs. the indicated group.

We studied the targeting relationship between miR-524-5p and CXCR7. The luciferase reporter vector CXCR7 3′UTR wild-type (WT) that we constructed contained the complementary sequence to miR-524-5p, and the CXCR7 3′UTR mutated (MUT) reporter plasmid was used as a control (Figure 4(a)). The results of the double luciferase reporter gene assay indicated that in the presence of the miR-524-5p mimic, the luciferase activity of CXCR7 3′UTR WT was reduced in HT-29 and Caco-2 cells. However, the miR-524-5p mimic did not reduce the CXCR7 3′UTR MUT luciferase activity in HT-29 and Caco-2 cells (Figures 4(b) and 4(c)). As shown in Figures 4(d) and 4(e), the expression of CXCR7 mRNA and protein in HT-29 cells transfected with miR-524-5p mimic was significantly reduced, and the expression of CXCR7 mRNA and protein in Caco-2 cells transfected with miR-524-5p inhibitor increased significantly.

### 3.5. MiR-524-5p Regulated Angiogenesis by Activating AKT/ERK Signaling in HT-29 Cells

**Figure 5:**
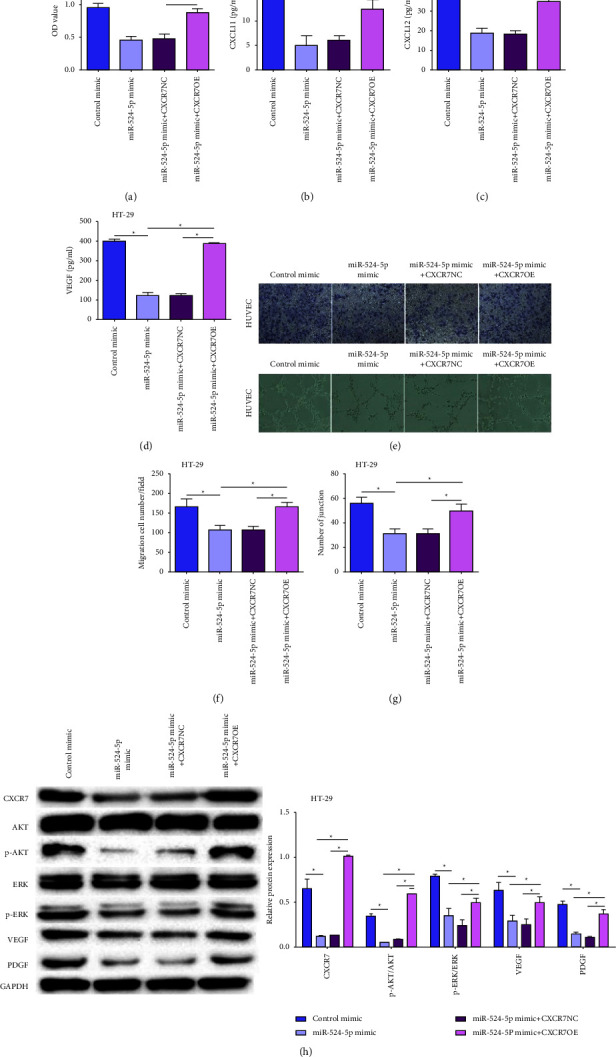
MiR-524-5p regulates angiogenesis via AKT/ERK signaling in HT-29 cells. (a) Cell proliferation was measured by the CCK8 assay in HT-29 cells after cotransfection. The release of CXCL11 (b), CXCL12 (c), and VEGF (d) in HT-29 cells after cotransfection. (e) Representative images of migration and tube formation assays. (f) Quantification of migration ability. (g) Quantification of angiogenic ability. (h) Protein levels of CXCR7, AKT, p-AKT, ERK, p-ERK, VEGF, and PDGF in HT-29 cells after cotransfection.

We manipulated the expression of miR-524-5p and CXCR7 in colon cancer cell lines to study their relationship with angiogenesis. The CCK-8 assay revealed that the reduced proliferation capacity of the miR-524-5p mimic could be reversed by overexpression of CXCR7 in HT-29 cells (Figure 5(a)). Simultaneously, the release of CXCL11, CXCL12, and VEGF could also be reversed (Figures 5(b)–5(d)). The co-culture of HT-29 cells transfected with miR-524-5p mimic and HUVEC cells reduced the number of migrating cells and the number of junctions, which was reversed by the overexpression of CXCR7 (Figures 5(e)–5(g)). Furthermore, CXCR7, VEGF, and PDGF expression and phosphorylation of AKT and ERK also increased after overexpression of CXCR7 in HT-29 cells transfected with miR-524-5p mimic (Figure 5(h)).

### 3.6. MiR-524-5p Regulated Angiogenesis by Activating AKT/ERK Signaling in Caco-2 Cells

**Figure 6:**
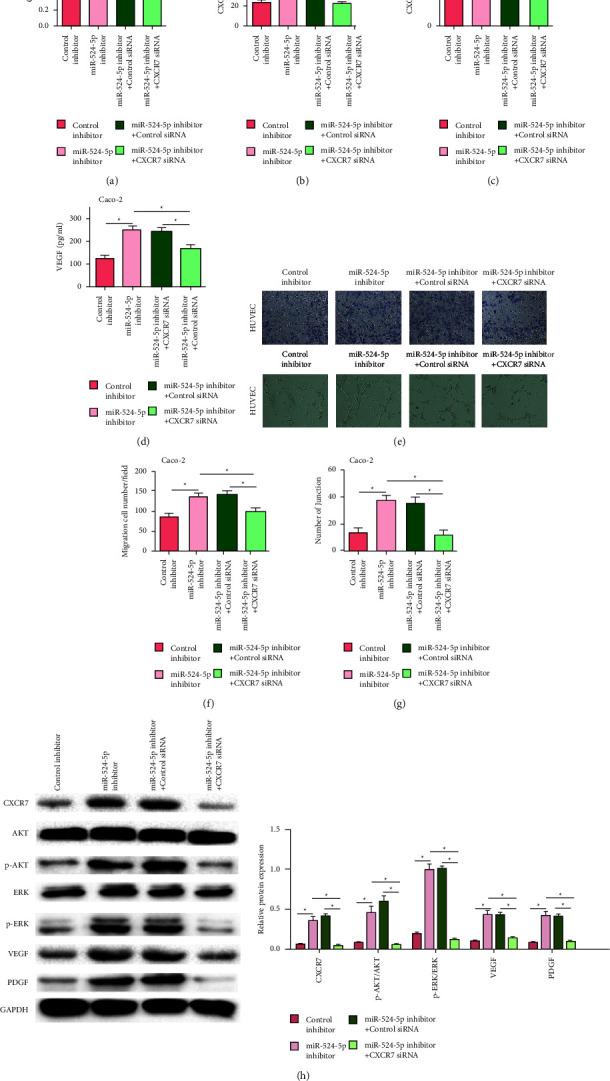
MiR-524-5p regulates angiogenesis by AKT/ERK signaling in Caco-2 cells. (a) Cell proliferation is measured by the CCK8 assay in Caco-2 cells after cotransfection. The release of CXCL11 (b), CXCL12 (c), and VEGF (d) in Caco-2 cells after cotransfection. (e) Representative images of migration and tube formation assays. (f) Quantification of migration ability. (g) Quantification of angiogenic ability. (h) Protein levels of CXCR7, AKT, p-AKT, ERK, p-ERK, VEGF, and PDGF in Caco-2 cells after cotransfection.

The CCK-8 results showed that the increase in proliferation capacity of the miR-524-5p inhibitor could be reversed by silencing CXCR7 expression in Caco-2 cells (Figure 6(a)), and at the same time, the release of CXCL11, CXCL12, and VEGF could also be reversed (Figures 6(b)–6(d)). Coculture of Caco-2 cells transfected with the miR-524-5p inhibitor and HUVEC cells increased the number of migrating cells and the number of junctions, which was reversed by the silencing of CXCR7 expression (Figures 6(e)–6(g)). Furthermore, CXCR7, VEGF, and PDGF expression and phosphorylation of AKT and ERK were also inhibited after silencing CXCR7 expression in Caco-2 cells transfected with the miR-524-5p inhibitor (Figure 6(h)).

### 3.7. Effects of MiR-524-5p on Tumor Growth in Vivo

**Figure 7:**
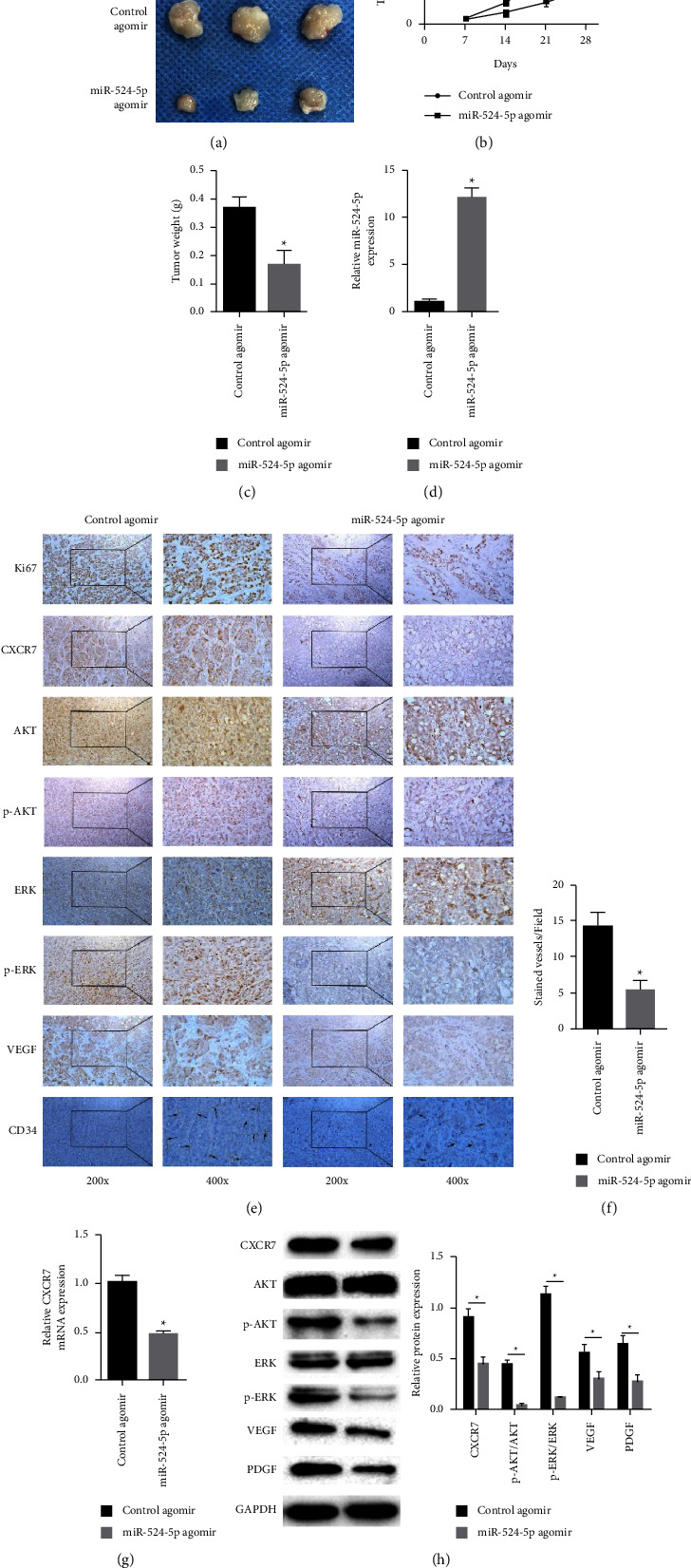
Effects of miR-524-5p on tumor growth in vivo. (a) An image of tumor tissue. (b) Tumor volume. (c) Tumor weight. (d) The relative miR-524-5p expression. (e) Tumor tissue sections were harvested for immunohistochemical staining of CXCR7, Ki67, VEGF, AKT, p-AKT, ERK, p-ERK, and CD34. (f) The count of CD34 microvessels reflected the MVD values. (g) The relative mRNA expression of CXCR7. (h) Protein levels of CXCR7, AKT, p-AKT, ERK, p-ERK, VEGF, and PDGF.

Finally, we injected HT-29 cells subcutaneously into BALB/c nude mice to study the effects of miR-524-5p overexpression on colon cancer growth (Figure S1). After injection, tumor size was tested every 7 days, and miR-524-5p agomir was injected into the tumor every 3 days. Nude mice were euthanized 28 days after tumor formation and weighed. The size of the transplanted tumor injected with miR-524-5p agomir was significantly reduced compared to the control group (Figure 7(a)). The size and weight of the transplanted tumor also decreased significantly (Figures 7(b) and 7(c)). The results of immunohistochemical staining confirmed that the expressions of Ki67, CXCR7, VEGF, CD34, p-AKT, and p-ERK in miR-524-5p agomir xenografts were significantly inhibited (Figures 7(e) and 7(f)). In addition, the expression of miR-524-5p in the miR-524-5p agomir xenograft was significantly increased (Figure 7(d)). In contrast, the expression of CXCR7 mRNA as well as the protein expression of CXCR7, VEGF, PDGF, p-AKT, and p-ERK was significantly suppressed (Figures 7(g) and 7(h)).

## 4. Discussion

In recent years, the role of miRNA in tumors has been extensively studied, including colon cancer [22]. Chen et al. showed that LINC00662 overexpression regulates colon cancer development through competitive binding to miR-340-5p [23]. Yan et al. showed that the overexpression of miR-182-5p in colon cancer cells significantly inhibited the carcinogenicity of SW620 cells and the angiogenesis and lymphangiogenesis of xenograft tumors in nude mice [24]. In this study, we examined changes in the proliferation, migration, and luminal formation of colon cancer cells (HT-29 and Caco-2) after forced up- or down-regulation of miR-524-5p. Overexpression of miR-524-5p inhibited the proliferation, migration, and luminal formation of colon cancer cells. The opposite was observed in the absence of miR-524-5p. We further tested the expression of VEGF, and the results showed that overexpression of miR-524-5p inhibited the expression of VEGF. In contrast, the lack of miR-524-5p increased the expression of VEGF. Altogether, these findings indicated that miR-524-5p was closely related to angiogenesis.

To further investigate the molecular mechanisms of miR-524-5p in colon cancer angiogenesis, we detected altered CXCR7 expression in transfected HT-29 and Caco-2 cells. Recently, the identification of CXCR7, formerly called the orphan receptor RDC1, was confirmed [18]. As a high-affinity receptor for CXCL12 and a low-affinity receptor for CXCL11 and CXCR7 serves as the key factor regulating cell survival, growth, and migration, rather than typical chemokine responses, such as calcium mobilization mediated by G protein-coupled receptors [21, 25]. Recently, several studies have shown the tumorigenic role of CXCR7 in polytype cancers, such as breast carcinoma and lung tumors, with stimulative growth and migration [18, 26]. Based on these findings, we analyzed CXCR7 expression after artificially engineering miR-524-5p expression. We found that CXCR7 expression was negatively regulated by miR-524-5p through target binding. Furthermore, knockdown of CXCR7 could decrease angiogenesis caused by loss of miR-524-5p. These results indicated that CXCR7 was an important downstream molecule of miR-524-5p.

Recent investigations have reported that CXCR7 can promote Akt phosphorylation, and the ERK pathway has also been found to play an important role in angiogenesis [27, 28]. In the present study, co-transfection with the miR-524-5p inhibitor and the CXCR7 siRNA was carried out in Caco-2 cells and co-transfection with the miR-524-5p mimic and CXCR7OE in HT-29 cells to investigate changes in angiogenesis, VEGF and PDGF production, and in Akt and ERK phosphorylation. Our data indicated that the angiogenetic ability of HUVECs was significantly enhanced following coincubation with the miR-524-5p inhibitor-transfectedCaco-2 cells, an effect that could be reversed by CXCR7 knockdown. Meanwhile, VEGF and PDGF production by miR-524-5p inhibitor-transfectedCaco-2 cells also increased and was accompanied by elevated phosphorylation levels of Akt and ERK. However, the angiogenetic ability of HUVECs was significantly reduced following incubation with miR-524-5p mimic-transfected HT-29 cells, and this effect could be reversed by CXCR7 overexpression. Meanwhile, VEGF and PDGF generation in transfected HT-29 cells treated with miR-524-5p mimic also decreased, which was accompanied by reduced phosphorylation levels of Akt and ERK. Therefore, we inferred that miR-524-5p/CXCR7 signaling regulated angiogenesis through AKT and ERK phosphorylation in colon cancer cells. Finally, the potential significance of increasing miR-524-5p expression was studied in transplanted tumor nude mice. Our findings provide evidence that the injection of miR-524-5p agomir reduced the expression of CXCR7, VEGF, PDGF, Ki67, CD34, and the levels of p-AKT and p-ERK, proving that miR-524-5p could inhibit the growth and angiogenesis of a colon cancer tumor. In conclusion, our results indicated that the miRNA-524-5p/CXCR7 axis regulated angiogenesis in colon cancer cells through the AKT and ERK pathways. CXCR7 will be a new target for future treatment and research in colon cancer.

## Data Availability

The datasets are available from the corresponding author upon reasonable request.

## Supplementary Materials

Supplementary Materials Figure S1: Schematic Diagram of Animal Research.
