# Supplementary material for: Inhibition of Angiogenesis by MiR-524-5p through Suppression of AKT and ERK Activation by Targeting CXCR7 in Colon Cancer Cells
Source: J Oncol. 2022 Nov 10;2022:7224840. doi: 10.1155/2022/7224840 (PMC9671741; doi:10.1155/2022/7224840)
Supplement: Supplementary Materials — Figure S1: Schematic Diagram of Animal Research. [file 7224840.f1.pptx]

## Slide 1
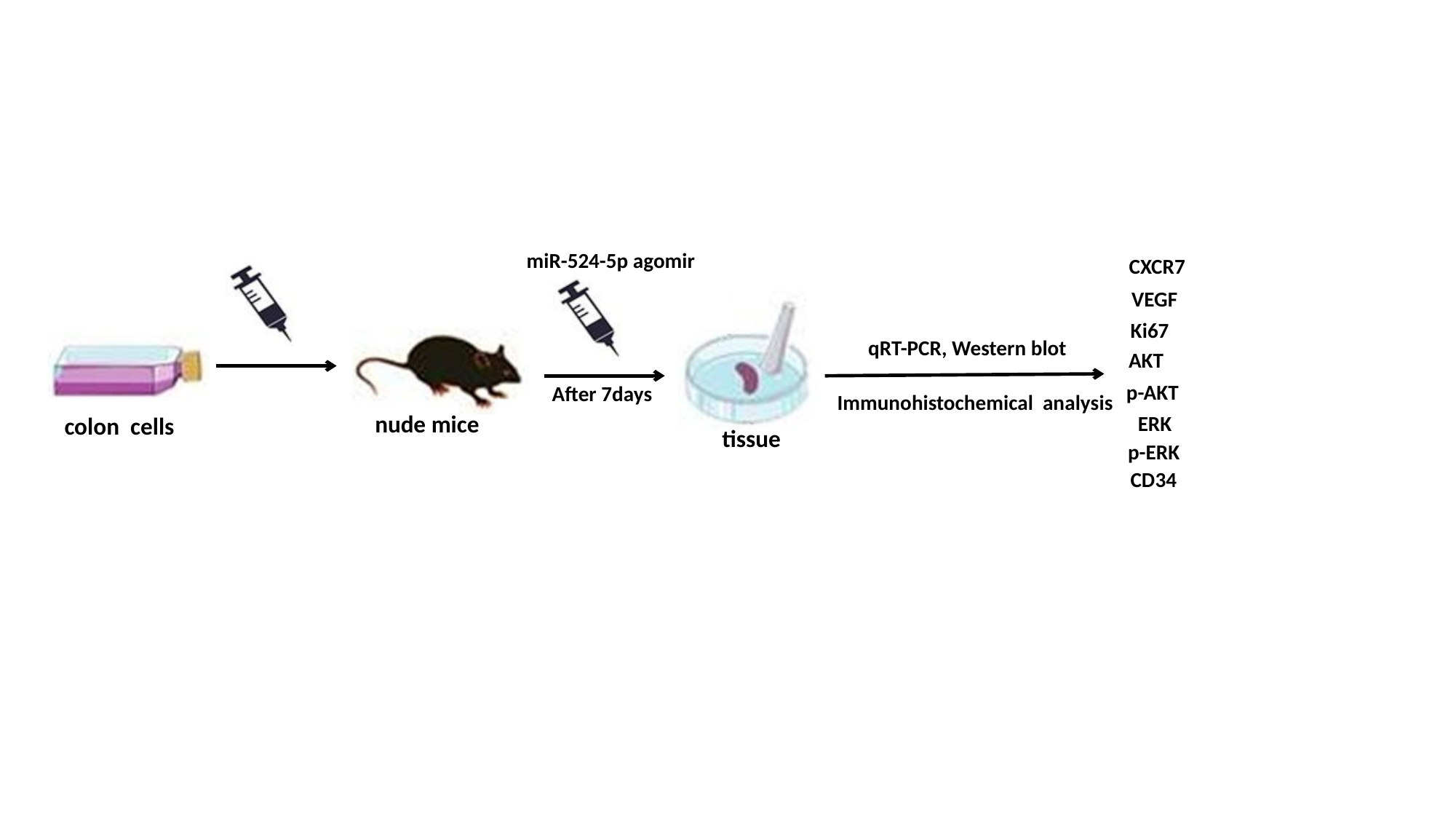

miR-524-5p agomir
CXCR7
VEGF
Ki67
qRT-PCR, Western blot
AKT
p-AKT
After 7days
Immunohistochemical analysis
nude mice
colon cells
ERK
tissue
p-ERK
CD34
